# *Trichoplax* genomes reveal profound admixture and suggest stable wild populations without bisexual reproduction

**DOI:** 10.1038/s41598-018-29400-y

**Published:** 2018-07-24

**Authors:** Kai Kamm, Hans-Jürgen Osigus, Peter F. Stadler, Rob DeSalle, Bernd Schierwater

**Affiliations:** 1 University of Veterinary Medicine Hannover, Foundation, ITZ Ecology and Evolution, Bünteweg 17d, D-30559 Hannover, Germany; 2 Bioinformatics Group, Department of Computer Science, and Interdisciplinary Center for Bioinformatics, University of Leipzig, Härtelstraße 16-18, D-04107 Leipzig, Germany; 3 Sackler Institute for Comparative Genomics and Division of Invertebrate Zoology, American Museum of Natural History, New York, New York, USA; 4 Yale University, Molecular, Cellular and Developmental Biology, New Haven, CT 06520 USA

## Abstract

The phylum Placozoa officially consists of only a single described species, *Trichoplax adhaerens*, although several lineages can be separated by molecular markers, geographical distributions and environmental demands. The placozoan 16S haplotype H2 (*Trichoplax* sp. H2) is the most robust and cosmopolitan lineage of placozoans found to date. In this study, its genome was found to be distinct but highly related to the *Trichoplax adhaerens* reference genome, for remarkably unique reasons. The pattern of variation and allele distribution between the two lineages suggests that both originate from a single interbreeding event in the wild, dating back at least several decades ago, and both seem not to have engaged in sexual reproduction since. We conclude that populations of certain placozoan haplotypes remain stable for long periods without bisexual reproduction. Furthermore, allelic variation within and between the two *Trichoplax* lineages indicates that successful bisexual reproduction between related placozoan lineages might serve to either counter accumulated negative somatic mutations or to cope with changing environmental conditions. On the other hand, enrichment of neutral or beneficial somatic mutations by vegetative reproduction, combined with rare sexual reproduction, could instantaneously boost genetic variation, generating novel ecotypes and eventually species.

## Introduction

The phylum Placozoa was discovered in 1883 by F.E. Schulze^1^ and so far consists of only one officially recognized species, *Trichoplax adhaerens*. Placozoans are irregular disc-shaped benthic animals of a few millimeters in diameter which crawl on hard substrates by ciliary movement or by expansions and contractions of their body (for review^2,3^). With only six somatic cell types identified to-date, placozoans exhibit the most simple morphology of a free-living metazoan^4–6^. Their simplistic bauplan and small genome (less than 100 Mb)^7^ has fueled the view that placozoans represent the closest extant surrogate of the last common metazoan ancestor, though molecular evidence for this traditional view is ambiguous^7–12^. In sharp contrast to the high degree of genetic variability between different lineages^13–15^, almost no variation on the basic placozoan bauplan has been observed, even at the ultrastructural level^16^. It seems plausible that the most simple design of placozoans does not allow major anatomical deviations. This is in marked contrast to all other non-bilaterian animals (cnidarians, sponges and ctenophores), which exhibit a high morphological diversity.

Understanding placozoan biology is further complicated by their enigmatic life cycle. Although Signorovich *et al*.^17^ reported molecular evidence for sex in local samples of a Caribbean lineage, lab cultures exclusively reproduce vegetatively (“asexually”). Under laboratory conditions, occasionally occurring oocytes are fertilized but the embryos cease development at the 128-cell stage at latest^18^. Moreover, *Trichoplax adhaerens* (16S haplotype H1, “Grell”)^13,19^, is most likely not the best placozoan lineage to use as a model system for the phylum. While *Trichoplax adhaerens* is rarely found in the field (e.g.^15^), the closely related *Trichoplax* sp. H2 (16S haplotype H2)^13^ is the most abundant and widely distributed placozoan lineage^13–15,20^. Furthermore, *Trichoplax* sp. H2 displays high reproductive rates even under suboptimal culture conditions. Hence, we suggest *Trichoplax* sp. H2 is a more suitable placozoan model system.

Species descriptions for placozoans are still impeded by their uniform morphology and the limited knowledge of their life cycle, bisexual reproduction, ecology and population dynamics (cf.^15^). At this point, species descriptions could be based only on genetic distances, for which no calibration is available. We thus avoid the systematic rank “species” to discriminate between H1 and H2 and use instead the descriptive terms “lineage” and “haplotype”, although both might represent distinct species. We here report on the *Trichoplax* sp. H2 genome and compare it to the *Trichoplax adhaerens* (H1) reference genome^7^. We find compelling evidence for increased diversifying natural selection between the lineages and a hybrid origin for one of the lineages.

## Results and Discussion

### Assembly and annotation statistics

The genome assembly of *Trichoplax* sp. H2 amounts to 94.9 Mb. Genome completeness was estimated by the presence of single copy eukaryotic or metazoan core orthologs using the CEGMA^21^ and BUSCO^22^ pipelines (Table 1). Furthermore, 98.5% of the genomic reads could be mapped to the genome assembly, 0.3% to an endosymbiont genome (see Methods) and 0.9% to the assembled mt-genome [Osigus *et al*., in prep.], making a total of 99.7%. Completeness was also assessed by mapping the de-novo assembled transcripts to the genome, yielding a mapping rate of 98.8%. Similar to the *Trichoplax adhaerens* genome^7^ the H2 genome shows a mean single nucleotide polymorphism (SNP) rate of 1% and an indel frequency of 0.1% (Supplementary Table S1). Multiallelic sites were found to be negligible, in the order of a few hundred, confirming that the multiple individuals used for library preparation were drawn from a population that exclusively reproduces by clonal division. The evidence-based gene prediction for *Trichoplax* sp. H2 resulted in 12,200 gene models with a mean size of 541 amino acids (Supplementary Table S1). Of these gene models, 81.4% had a Swiss-Prot hit, 94.8% yielded an InterProScan^23^ result (see also Supplementary Fig. S1) and 69.6% were assigned a gene ontology (GO) term.

**Table 1:** Assembly statistics of the *Trichoplax* sp. H2 genome and the reference genome.

Assembly	Assembly size (≥2 kb)	Scaffolds (≥2 kb)	N50	Largest Scaffold	# Ns	CEGMA completeness^b^	BUSCO completeness^b^
*Trichoplax* sp. H2	94.9 Mb	1,128	376.3 kb	1.66 Mb	44.8 kb	95.2/97.2%	91.3/95.6%
*Trichoplax adhaerens* ^*a*^	104.6 Mb	703	5.98 Mb	13.3 Mb	10.8 Mb	93.6/95.6%	90/94.7%

^a^Fragments of an endosymbiont genome were removed from the reference genome (see Methods).

^b^Completeness based on unfragmented only/plus fragmented gene models.

### The two *Trichoplax* genomes are closely related

**Figure 1:**
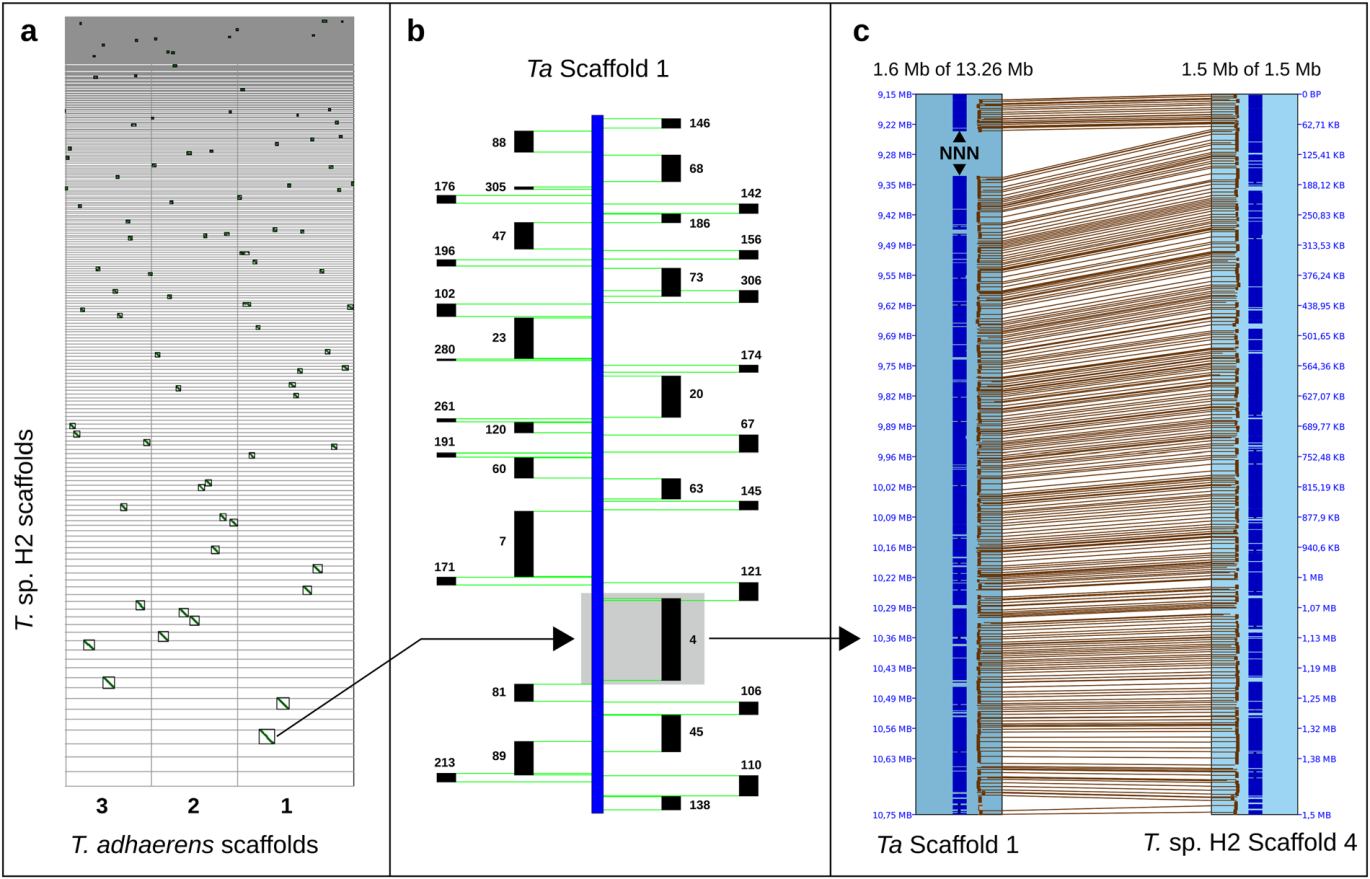
Synteny analyses. The *Trichoplax* sp. H2 genome shows almost complete synteny to the reference genome, up to the resolution the contiguity of the scaffolds can provide. (**a**) Syntenic dotplot based on collinear gene pairs between the three largest scaffolds of the reference genome and the genomic scaffolds of *Trichoplax* sp. H2. (**b**) Block view of the H2 scaffolds syntenic to scaffold 1 of *Trichoplax adhaerens* (**c**) *Trichoplax* sp. H2 scaffold 4 mapped to *Trichoplax adhaerens* scaffold 1 showing collinear gene pairs. Scaffolds below 50 kb were excluded in the figures for clarity.

At the nucleotide level both genomes align to each other with an overall identity of 99.1%. The total alignment length amounts to 92.2 Mb in both genomes, equivalent to a coverage of 97.2% for the H2 and 98.2% for the H1 genome. If the two genomes are aligned using gene models (Fig. 1, Supplementary Fig. S2), they show almost complete synteny: After condensing tandem duplicated genes, the two genomes could be aligned with 8,970 collinear gene pairs and along the larger H2 scaffolds no obvious breakpoints or shuffling of gene order could be detected. Because at least five gene pairs are required for a syntenic alignment, smaller scaffolds and those harboring many tandem duplicated genes are excluded in this approach. Differences between predicted gene models further complicate correct identification of collinear gene pairs because they may occur even between similar genomes as a result of the different transcriptomes given as evidence (Supplementary Table S1) and because of the multidomain structure of eukaryotic proteins^24^. However, 296 scaffolds of *Trichoplax* sp. H2 (84.7 Mb in total) are clearly syntenic, which amounts to about 90% for both genomes. Even the genomic paired-end reads of *Trichoplax* sp. H2 can be aligned to the reference genome, though with a lower alignment rate (96.6%) and resulting in a higher polymorphism rate (1.15% SNP, 0.11% indel; see also Supplementary Fig. S3 and Table S1).

A similar picture shows the comparison at the protein level (Supplementary Fig. S3): The two lineages share 10,030 orthologous clusters of which 9,900 are single-copy gene clusters. The sequence clustering yielded a certain proportion of singletons for both, but many genes which fall into this category contain repetitive domains like tetratricopeptide, EGF-like or Leucine-rich repeats, which may result in the prediction of slightly deviating gene models between both genomes, also affecting the correct identification of orthologs. In addition, several genes fail to find a match because of incomplete assemblies/missing data. For example, we found ten scaffolds of at least 48 kb in the *Trichoplax* sp. H2 assembly which have no counterpart in the reference genome and amount to 1.4 Mb (Supplementary Table S1). Novel placozoan genes encoded on these scaffolds include, for example, the protein kinase A catalytic subunit and the microprocessor DGCR8, both of which are key components of crucial cellular pathways. The comparison of single-copy orthologs showed that 28% are completely identical and 77.5% have identities of ≥99%.

Analyses of repeats showed that both genomes harbor an almost identical repeat content of 6.6%/6.7% for *Trichoplax* sp. H2 and *Trichoplax adhaerens,* respectively (Supplementary Table S2). This amount is higher than previously reported (2.8%) for *Trichoplax adhaerens*^25^ but the majority of the additional elements are unclassified interspersed repeats identified via lineage-specific libraries. Still this amount of repetitive elements is much smaller than those reported for other basal metazoans like *Aiptasia pallida* (26%), *Acropora digitifera* (13%)^26,27^ or for most bilaterians^28^. We also detected a slightly higher amount of DNA transposons (0.6% vs 0.5%) than previously described of which most belong to the Ginger family of cut-and-paste transposases (0.3%)^29^. While Non-LTR retrotransposons are essentially absent in both genomes (0.04%), we found a slight difference in the amount of LTR retrotransposons (0.11% vs 0.16%) which is solely based on the Ngaro family (0.003% vs 0.04%). However, homology-based searches have previously shown that most transposable elements in *Trichoplax* are probably inactive^25^. In line with this, the here identified repetitive elements are generally small (less than 200 entries ≥1 kb). Additionally, scanning the translated ORFs, using HMM profiles of all Pfam entries for reverse transcriptases, integrases or transposases, identified only 9 (12 in *T. adhaerens*) sequences satisfying a profile’s gathering threshold (Supplementary Table S2). In conjunction with the only marginal differences in repeat content between the two genomes, we thus conclude that transposable elements currently are not significantly involved in shaping the genomic landscape of the two *Trichoplax* haplotypes which is in accordance with the high level of sequence similarity and synteny between them.

### Genes show evidence for positive selection

To assess the amount of divergence and evaluate how natural selection has shaped the two genomes, synonymous and non-synonymous substitution rates between collinear gene models were estimated. These values have to be viewed cautiously because the assemblies constitute a non-phased consensus of two closely related diploid organisms with a high polymorphism rate (Supplementary Fig. S3, Table S1, Srivastava *et al*.^7^). Nevertheless, if interpreted tentatively the data are useful to uncover regions of divergence. The mean synonymous substitution rate between collinear pairs was found to be 0.024 which indicates a rather recent divergence of the two lineages and/or a low effective population size, according to neutral theory^30^. For example, the average synonymous substitution rate within *Branchiostoma belcheri* was estimated to be three times larger^31^ while the rate within human or chimpanzee is about one third^32^ mirroring the substantial differences in effective population sizes.

Conversely, the corresponding dN/dS value is 0.37 and thus much higher than within *Branchiostoma belcheri* (0.067–0.089)^31^, *Danio rerio* (0.142)^33^, *Ciona savigny* (0.07)^34^, or hominids (0.23)^32^. This observation most likely relates to the low synonymous substitution rate between the two placozoan genomes, but also indicates that some genes have diverged between the two lineages as a result of positive selection.

**Figure 2:**
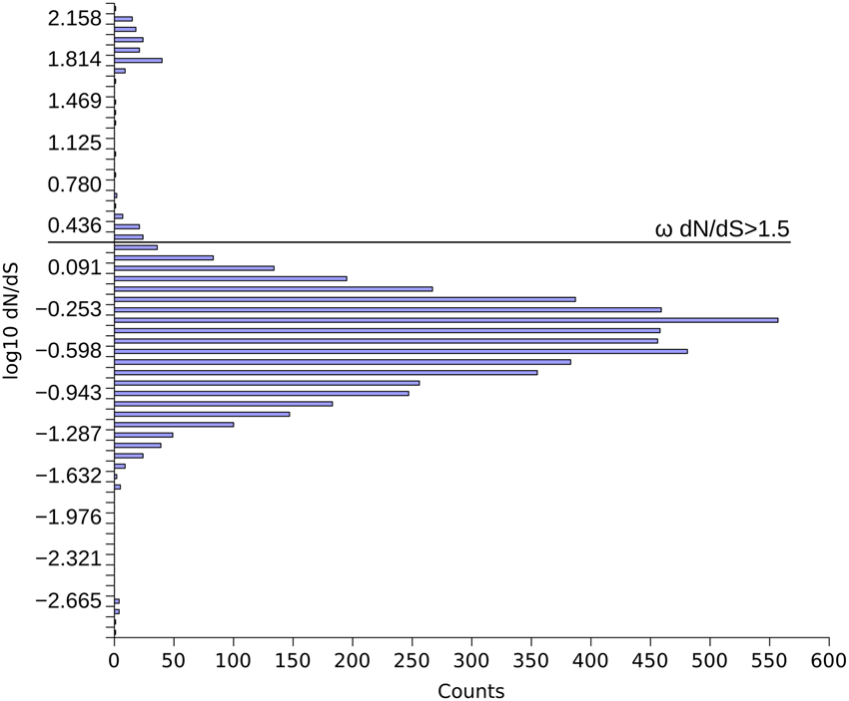
dN/dS distribution of collinear gene pairs between *Trichoplax adhaerens* and *Trichoplax* sp. H2. The distribution shows that purifying selection dominates, but some genes show evidence of positive selection. Of the initial ≈9,000 pairs, those were removed that showed either dS = 0, dN = 0, or saturated values of either two (≥2).

Looking at the dN/dS values of individual gene pairs, the distribution of the collinear pairs dN/dS (Fig. 2) shows that purifying selection is acting on the majority of placozoan genes. Some genes show evidence of positive selection, however. In particular we identified about 230 genes with dN/dS ratios above 1.5 (examples are shown in Supplementary Table S3). The most notable of these are a putative homolog of the transcriptional modulator SMAD6 and two transcription factors of the homeobox NK family, DBX and NK6^35^, whose orthologs in Metazoa are usually highly conserved.

### The two lineages share at least one identical allele in most loci

**Figure 3:**
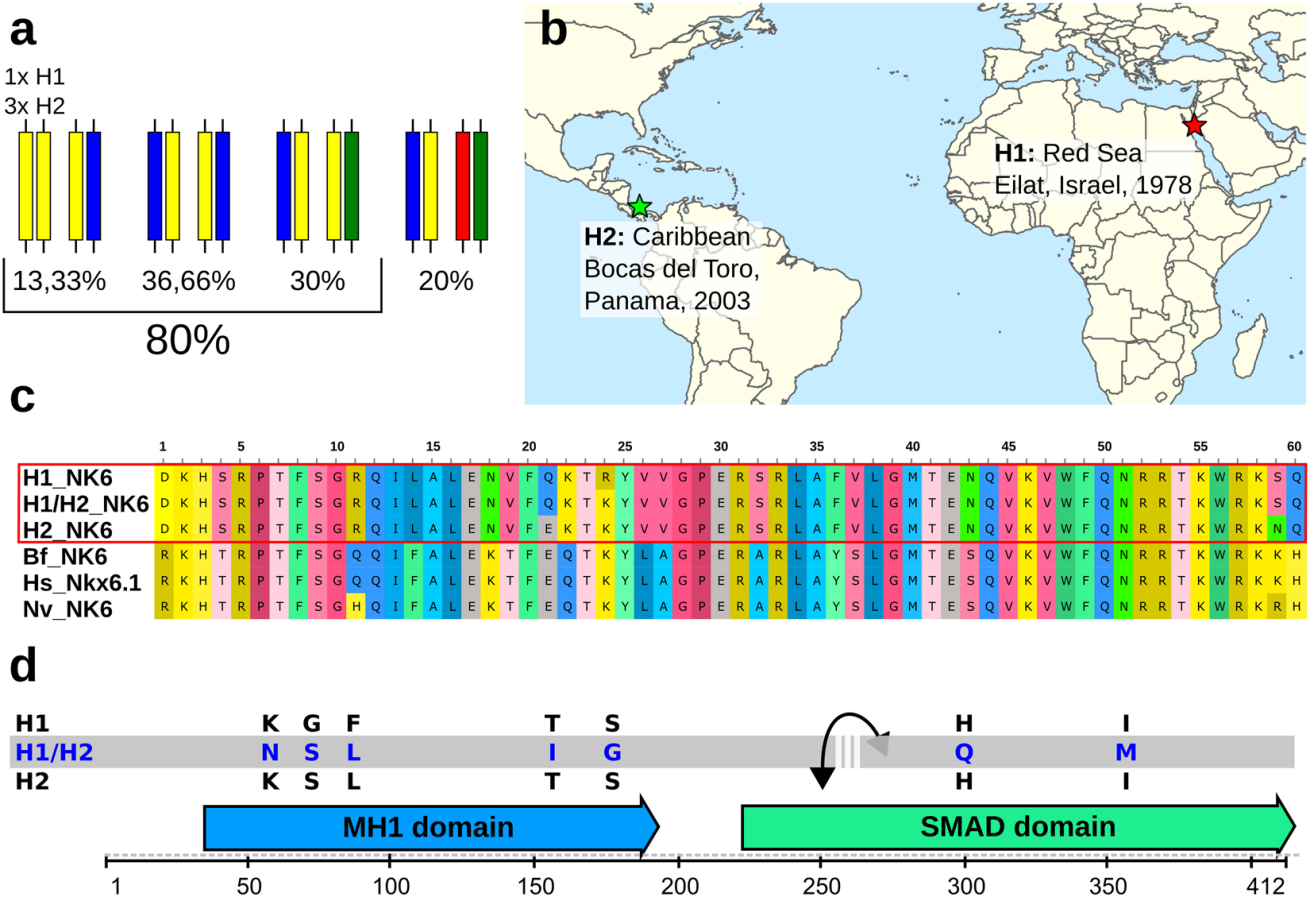
Hybrid speciation in Placozoa? (**a**) Allele distribution between H1 and H2 observed in 30 phased coding sequences (CDS). In 80% of investigated CDS the two haplotypes share at least one allele. (**b**) Sampling site and year of the two haplotypes. (**c**,**d**) Two cases of developmental transcriptional regulators in which both haplotypes share one identical allele and the second allele shows a high deviation that is usually not observed within a species. (**c**) In the case of the NK6 homeodomain we observed two amino acid substitutions within H2, one within H1 and three between the unshared alleles. Note that between *Branchiostoma* and human there are zero substitutions and even between the cnidarian *Nematostella* and human there are only two. (**d**) Similarly, the placozoan SMAD6 ortholog shows high intraindividual variation but both haplotypes share one allele. Shown are the amino acid substitutions between the shared/unshared alleles. The interrupted grey bar in d indicates that the phasing of the transcript resulted in two phased blocks. H1 = *Trichoplax adhaerens*, H2 = *Trichoplax* sp. H2, Bf = *Branchiostoma floridae*, Hs = *Homo sapiens*, Nv = *Nematostella vectensis*.

Because both genome assemblies represent a consensus of the respective two sets of homologous chromosomes, we further examined the possibility that some of the differences between the two genomes can be attributed to haplotypic variation. To test this hypothesis, we manually phased 30 coding sequences (CDS), which showed at least one substitution between the two lineages. Phasing was done by mapping lineage specific RNA-seq reads against the CDS aided by mapping the genomic reads (trace files in the case of *T. adhaerens*)^7^ against the annotated genomes. The 30 CDS amount to 29 kb, showed 254 single nucleotide polymorphisms (SNPs) altogether and could be phased into 37 blocks. The corresponding loci are located on 10 and 28 different scaffolds in the assemblies of *Trichoplax adhaerens* and *Trichoplax* sp. H2, respectively. The results (Fig. 3a, Supplementary Fig. S4, Table S4) show that both *Trichoplax* genomes share at least one identical allele in the coding sequence of 80% of the investigated loci. We encountered only 6 loci with four different alleles (20%) and the respective closest two alleles between the two altogether harbor only 8 substitutions. Since we investigated only non-identical consensus CDS, the ratio of loci without a shared allele must be even lower than 20%.

### The pattern of allele distribution suggests a single interbreeding event, a long period without successful sexual reproduction and fixation of somatic mutations

The number of shared alleles is only possible if *Trichoplax adhaerens* and *Trichoplax* sp. H2 have either a parent-F1 or a two-sibling relationship and both terminal lineages have not engaged in sexual reproduction since their origin. Otherwise recombination during meiosis would have markedly dropped the rate of identical alleles. We can eliminate self-fertilization in a clonal population as a possibility, because this would eventually lead to complete homozygosity, which apparently is not the case given the high rate of polymorphisms in both haplotypes.

Since the genetic identity, deduced from the ratio of shared alleles, deviates from 50%, a two-sibling relationship appears more likely, because the F1 generation should share half of their genes with each parent (there is no evidence for heterochromosomes in placozoans^36^). However, at the rare loci without a shared allele, both lineages possess one allele each which differ only by point mutations and are much more related to each other than to the respective second alleles. It thus seems likely that these point mutations represent somatic mutations that have become fixed in the population. This propagation and eventual fixation of somatic mutations has been already described in Cnidaria, especially colonial anthozoans, which also reproduce vegetatively during some stages of their life cycle and also lack a strict germline segregation^37^. While germline segregation leads to the loss of all somatic mutations with a single round of sexual reproduction, lack of it allows the enrichment of mutated cell lines by chance, intrasomatic competition and numerous vegetative fissions. This process goes on, until an animal arises in which all cells carry the mutation, provided that the mutation is either neutral or beneficial. Considering the different sampling dates (25 years apart) and locations (12,000 km apart) of the two placozoan lineages (Fig. 3b)^15^, we conclude that their separation dates back at least long enough to explain the observed pattern. Although we still have only limited knowledge about the passive oceanic dispersal of placozoans by pelagic swarmers, by adult animals attached to floating objects or by shipping traffic (cf. Pearse & Voigt^38^), the two lineages must have once occurred sympatric and were dispersed afterwards. About the time-frame we can only speculate but the large distance between the sampling locations emphasizes the minimal separation time given by the different sampling dates.

### The two placozoan lineages show substantial intra- and interspecific allelic variation

We emphasize that the observed allele differences within and between haplotypes are substantial in several cases and would separate lineages at least at the species level in other taxa (Fig. 3c,d): For example, the shared allele of the placozoan SMAD6 homologue shows 5 or 3 amino acid (AA) substitutions in the N-terminal DNA-binding domain (MH1) and 2 in the protein-protein interacting MH2 (SMAD) domain compared to the respective second alleles in *Trichoplax adhaerens* and *Trichoplax* sp. H2. The aforementioned homeobox gene NK6 is also represented as one shared allele and two which differ. Looking only at the AA substitutions within the (usually highly conserved) homeodomain, one substitution is present between the two alleles of *Trichoplax adhaerens* and two within *Trichoplax* sp. H2, while the respective second alleles deviate by three AA substitutions. A comparison to other metazoans further highlights this surprising finding. The human Nkx6.1 shows not a single substitution in the homeodomain compared to its ortholog NK6 in the cephalochordate *Branchiostoma*. Even NK6 of the cnidarian *Nematostella* deviates from the latter by only two AA substitutions. We are not aware of any other cases of metazoan homeobox genes showing non-synonymous substitutions in any homeodomain within the same species or even the same individual. The placozoan NK6 is a clear ortholog of the bilaterian NK6^35^ and all alleles are expressed. Hence, it is unlikely that the gene represents a pseudogene, but its function in placozoans, and that of the different alleles, is yet unknown.

### Possible implications for reproductive strategies, ecological adaptation and speciation in Placozoa

In addition to the observed pattern of allele distribution, both lineages also harbor divergent mitochondrial genomes [Osigus *et al*., in prep.]. Hence, we suggest that one of the two lineages is the result of a “hybridization” between the other and a third, yet unknown, placozoan lineage that contributed the second set of alleles and the second mitochondrion. We can exclude with near certainty that the hybridization had occurred in our lab because the variant analyses of both genomes (Srivastava *et al*.^7^; this study) confirmed that the hundreds of individuals used for the isolation of genomic DNA were drawn from a clonal population. The single founder animal of the respective clonal lineage^13,19^ therefore must have already possessed all point mutations mentioned above. Otherwise we would observe these mutations as multiallelic sites because they arise in single cells of single individuals.

Since this relationship is still recognizable after at least 25 years of separation, we also conclude that wild populations of these placozoan lineages are the result of decades of vegetative reproduction and that mating with clonal conspecifics is rarely successful, probably because the resulting lower levels of heterozygosity would negatively affect an individual’s fitness. An alternative explanation would be that the two lineages have lost the ability for sexual reproduction as a result of “hybridization”. In this sense, meiosis or other regulative pathways essential for sexual reproduction (e.g. formation of eggs) could have become negatively affected by the presence of two distinct genomes in the cells^39^. However, oocyte formation, maturation and fertilization has been unambiguously demonstrated in both lineages^18^.

It thus seems more likely that the high level of heterozygosity in the two placozoans constitutes a buffer against deleterious mutations and this could also be the underlying cause why embryonic development in placozoan lab cultures has never been observed beyond the 128-cell stage^18^. Most likely, sexual reproduction among clonal individuals frequently leads to inviable embryos as a result of homozygous deleterious mutations. In the phased *Trichoplax adhaerens* CDS we detected one striking example for this scenario: A bone morphogenic protein (BMP7) related gene is present as four different alleles in H1 and H2 and the most related two alleles between them differ in only one substitution. This substitution introduces a premature stop codon in the H1 transcript cutting off the last third of the C-terminal TGF-beta domain, most probably leading to a nonfunctional protein (Supplementary Fig. S4, Table S4). The second allele, however, encodes a complete domain and could complement the function of the disrupted BMP.

Mating with a different (but related) lineage could thus also serve to escape Muller’s Ratchet in the long run and to overcome the accumulation of too many deleterious mutations by continuous asexual reproduction^40^. While it is likely that most recombinants will be unfit, some may receive a favorable allele combination that simultaneously boosts genetic variance and possibly enables adaptation to different environmental conditions^41^, eventually leading to speciation. We have to emphasize that this scenario cannot be generalized to the entire phylum since Signorovich *et al*.^17^ detected evidence for continuous sexual reproduction in a Caribbean population of a different placozoan lineage. Obviously, different placozoan lineages use different reproductive strategies to cope with their specific needs.

## Methods

### Animal material

The placozoan lineage *Trichoplax* sp. H2 “Panama” has been collected in the Caribbean, Bocas del Toro, Panama in 2003^13,42^. *Trichoplax adhaerens* (“Grell”, H1) originates from the Red Sea, Eilat, Israel^13,19^ and is the same lineage that has been used for the *Trichoplax* genome sequencing in 2008^7^. All placozoan lineages are cultured as clonal strains in our lab as previously described^14^.

### Genome and transcriptome sequencing

Prior to genomic DNA isolation the animals were transferred to a clean glass petri dish, starved at least for two days and washed several times with clean artificial seawater (ASW). DNA from *Trichoplax* sp. H2 was extracted using a standard phenol-chloroform nucleic acid extraction protocol^43^ with subsequent RNase digest. For RNA isolation the animals were starved for only one day to minimize the influence of starvation on transcription. Total RNA from *Trichoplax* sp. H2 and *Trichoplax adhaerens* was extracted using a standard phenol-chloroform nucleic acid extraction protocol^43^ with subsequent DNase digest.

The *Trichoplax* sp. H2 genomic DNA paired-end library had a targeted insert size of 500 bp and was prepared following the Illumina protocol “Preparing Samples for Sequencing Genomic DNA” protocol (Part # 1003806 Rev. B, March 2008) and sequenced on an Illumina HiSeq 2500 (2 × 150 bp) at the Yale Genome Center (Connecticut, USA). This sequence run resulted in 56.4 million paired-end reads.

RNA-Seq libraries for *Trichoplax* sp. H2 and *Trichoplax adhaerens* with a targeted insert size of 150–200 bp were constructed following the Illumina protocol “Preparing Samples for Sequencing of mRNA” (Part # 1004898 Rev. A, September 2008) and sequenced at the Yale Genome Center (Connecticut, USA) on an Illumina HiSeq 2500 instrument (2 × 75 bp). This resulted in 150.7 and 64.3 million paired-end RNA-Seq reads for *Trichoplax adhaerens* and *Trichoplax* sp. H2, respectively.

The paired-end reads were inspected with FastQC^44^ and quality trimmed with Trimmomatic 0.33^45^ (RNA-Seq reads were quality trimmed within the Trinity^46^ pipeline; see below).

### *Trichoplax* sp. H2 genome assembly

The following assembly pipelines for de-novo assembly were initially tried: SGA 0.10.13^47^, dipSPAdes 3.5^48^, Platanus 1.2.1^49^ and MaSuRCA-2.3.2^50^. The MaSuRCA assembly yielded the best assembly in terms of contiguity (N50) and completeness (estimated by CEGMA^21^). MaSuRCA was run with default parameters except for the CA_PARAMETER utgErrorRate = 0.03, which was added to the pipeline parameters to better merge haplotypes.

Since the primary assembly with MaSuRCA revealed a high relatedness to the *Trichoplax* reference genome, it was subjected to a secondary assembly with AlignGraph^51^ using the reference genome as guidance^7^. Briefly, in the guided secondary assembly with AlignGraph the de-novo generated scaffolds are aligned to the reference and the paired-end reads are mapped to the assembled scaffolds and to the reference. This results in a paired-end multi-positional de Bruijn graph from which the scaffolds are extended if possible. AlignGraph was provided with the MaSuRCA generated scaffolds of at least 2 kb length and run with the standard parameters suggested for paired-end insert size (500 bp) and single read length (150 bp). The mitochondrial genome of *Trichoplax* sp. H2 was assembled separately and will be published elsewhere. Therefore the mitochondrial scaffolds were also removed from the MaSuRCA assembly prior to the secondary assembly.

### Redundancy and contaminant removal

Contaminant sequences were detected by blasting all scaffolds below 10 kb and those showing deviations in GC content against the NCBI bacterial reference genomes and non-redundant nucleotide collection. Since both *Trichoplax* genomes have a GC content around 32.7%, all scaffolds below 30% and above 35% GC were considered to deviate.

Redundant scaffolds were detected by an all-versus-all search with BLASTN^52^. A scaffold was considered redundant and removed if it was enveloped by a larger scaffold, showed at least 98% identity and its sequence was covered 80% or more by the larger scaffold. In rare cases overlaps were detected and these were joined together if the overlap (1) was at least 1,000 bp supported by a 10x or higher read coverage (2) showed at least 99% identity (3) mismatches could be attributed to haplotypic variation by the mapped reads (4) BLASTN revealed no other, conflicting, alignment.

As was done for the H2 genome assembly, the *Trichoplax adhaerens* reference genome was also cut-off below 2 kb for later comparative purposes. The rationale for doing this in both genomes was that the fraction of smaller scaffolds usually contain many contaminating sequences and are of low informative value because of incomplete genes. This approach was confirmed by an initial gene prediction of the *Trichoplax adhaerens* genome which revealed that of the roughly 200 predicted genes in the 662 scaffolds below 2 kb, more than 70% were clearly of non-metazoan origin (mostly bacterial as determined by BLASTP against the NCBI non-redundant protein database), while the remaining had either *Trichoplax*-only hits and/or were highly fragmented.

### Endosymbiont genome removal

After release of the *Trichoplax* reference genome, bacterial genes have been detected in the assembly. While some of them reside on host chromosomes, the remainder clearly belong to an incomplete and fragmented bacterial genome of a rickettsial endosymbiont^53^. Because these endosymbiont sequences show only weak similarity to genomes deposited in databases, their identification in the *Trichoplax* sp. H2 assembly was carried out in a stepwise fashion: (1) a rickettsiales protein set from UniProt (*Rickettsia bellii*, endosymbiont of *Acanthamoeba* sp. UWC8 & UWC36, *Midichloria mitochondrii and Wolbachia pipientis*) was blasted against the proteins of a preliminary gene prediction (e-value cutoff 1e-100; annotation and prediction details see below). (2) Positive protein hits were blasted against the NCBI non-redundant protein database to verify their bacterial origin (3) a corresponding “positive” scaffold was considered as likely of endosymbiont origin if all of its predicted genes’ best blast hits showed a preponderance to bacterial proteins and thus most probably contains not a single eukaryotic gene (4) scaffolds containing only bacterial genes were found to have a GC content around 27% and subsequently all scaffolds of 30% GC or below were considered as likely belonging to the endosymbiont genome.

Eventually, these candidate scaffolds were considered as clearly endosymbiont scaffolds if: (I) They contained not a single eukaryotic gene (II) GC content was 30% or below (III) Reads mapped to these scaffolds revealed no sign of haplotypic variation (IV) Read coverage was significantly below the expected coverage of 80x (e.g. around 15x for the bacterial genome), or significantly higher (e.g. likely plasmids).

The H2 endosymbiont scaffolds were subsequently used to identify and remove endosymbiont sequences in the *Trichoplax* reference genome. The H2 endosymbiont scaffolds were blasted against the reference genome and scaffolds showing 80% or more identity were removed. To further confirm their bacterial origin, the corresponding proteins from a preliminary gene prediction (see below) were blasted against NCBI’s non-redundant protein database. Altogether, 50 scaffolds amounting to 215 kb (including 73 kb of Ns) were removed from the reference genome.

### Assembly completeness estimation

Assembly completeness for the H2 genome was estimated by mapping the 150 bp paired-end reads against the H2 assembly, the endosymbiont assembly and the mitochondrial genome^54^ [Osigus *et al*., in prep] with BWA MEM^55^ and calling the mapping rate with Samtools 1.2^56^. The completeness was also assessed by estimating the presence of core eukaryotic and core metazoan genes using CEGMA v2.5^21^ and BUSCO v1.1b1^22^, respectively. BUSCO was used along with Augustus 3.0.3^57^ and full optimization of gene model parameters. Furthermore, the mapping rate of the de-novo assembled transcripts (see below) to the genome of *Trichoplax* sp. H2 was assessed using BLAT^58^.

### Transcriptome assembly

The transcriptomes of *Trichoplax adhaerens* and *Trichoplax* sp. H2 were assembled de-novo with Trinity v2.0.6^46^. Trinity was run with the –Trimmomatic option for quality trimming and the parameter –jaccard_clip to minimize fusion transcripts. Protein coding genes were predicted from the transcripts using TransDecoder v2.0.1^59^. BLASTP hits against Swiss-Prot and positive hits of a scan with HMMER (v3.1^60^) against the PFAM database were used to support the TransDecoder prediction. The transcriptomes were also assembled using a genome-guided approach with TopHat2^61^ and Cufflinks^62^.

Transcript quantification of the de-novo assembled transcriptomes was carried out with RSEM v1.2.28^63^ using the accompanying script of the Trinity pipeline.

### Repeat content and classification

For repeat identification and classification RepeatMasker (version open-4.0)^64^ was used with a lineage-specific repeat library that was added to all species’ entries of the RepBase library (release 20150807). The lineage-specific repeat library was created using RepeatModeler (version open-1.0.8)^65^. The resulting repeat consensi were searched for conserved domains using NCBI’s conserved domain database and consensus sequences containing positive hits for eukaryotic domains were removed from the lineage-specific library. Repetitive elements in the genomes were identified with RepeatMasker and classified using the accompanying script buildSummary.pl.

To search for conserved domains related to transposable elements, the repeat sequences from the RepeatMasker output were extracted with gffread 0.9.8c^66^ and sequences below 300 bp were discarded. All open reading frames with a minimum size of 150 bp and the respective amino acid translations were then extracted using getorf of the EMBOSS suite (v6.6.0.0^67^). The resulting protein sequences were scanned using HMMER with all Pfam entries for reverse transcriptases, transposases and integrases. The threshold for reporting a positive hit was a profile’s gathering cutoff.

### Gene prediction and annotation

For gene prediction and annotation of the *Trichoplax* sp. H2 and the reference genome the evidence-based Maker annotation pipeline (v2.31.8) was used^68^ along with the gene predictors Augustus 3.0.3^57^ and eukaryotic GeneMark.hmm (part of GeneMark-ES Suite 4.21)^69^. Augustus was trained specifically for both genomes by submitting the respective de-novo assembled transcripts to the training pipeline WebAugustus^70^. Lineage-specific model parameters for GeneMark were created using Genemark-ET (GeneMark-ES Suite 4.21)^71^ provided with the intron coordinates generated by TopHat2 in the course of the genome-guided transcriptome assembly.

Evidence given to Maker consisted of the respective de-novo and genome-guided assembled transcripts from Trinity and Cufflinks. Additional evidence was a custom protein dataset including all Swiss-Prot entries from *Homo sapiens* and Protostomes and all UniProt entries for *Nematostella vectensis*, *Amphimedon queenslandica*, *Trichoplax adhaerens* and *Strongylocentrotus purpuratus*. Furthermore, TransDecoder predictions from all placozoan transcriptomes available in our lab were added and the whole protein dataset was reduced to 98% non-redundancy with CD-HIT^72^.

For repeat masking within the Maker pipeline, RepeatMasker was used with all species in RepBase, together with the lineage-specific libraries from above. Additionally, the Maker accompanying RepeatRunner was used to identify and mask TE-elements in protein space. Soft-masking for simple repeats was used to allow the extension of evidence sequences alignments into low-complexity regions of the genomes by BLAST. Gene prediction statistics were calculated with Eval v2.2.8^73^.

Functional annotation of the predicted proteins was carried out using InterProScan (5.19–58.0)^23^ with the following analyses: CDD-3.14, SignalP_EUK-4.1, PIRSF-3.01, Pfam-29.0, SignalP_GRAM_POSITIVE-4.1, TMHMM-2.0c, PRINTS-42.0, ProSiteProfiles-20.119, PANTHER-10.0, Coils-2.2.1, Hamap-201605.11, ProSitePatterns-20.119, SUPERFAMILY-1.75, ProDom-2006.1, SMART-7.1, SignalP_GRAM_NEGATIVE-4.1, Gene3D-3.5.0 and TIGRFAM-15.0. Annotation of predicted proteins also included BLASTP searches against Swiss-Prot (cutoff e-value 1e-5) and KEGG pathway mapping using KAAS^74^.

### Variant calling

The quality trimmed genomic Illumina PE reads of *Trichoplax* sp. H2 were mapped to the *Trichoplax* sp. H2 and the *Trichoplax adhaerens* reference genome using BWA MEM^55^ and the resulting alignment map files were further processed with Samtools 1.2^56^, GATK 3.4^75^, Picard-Tools 1.135^76^ and Bcftools 1.2^56^. Briefly, read pairing information and flags were cleaned and the reads sorted from name into coordinate order with Samtools. To reduce the number of miscalls of indels, the raw gapped alignment was realigned with the GATK Realigner which optimizes read alignment around indels. PCR and optical duplicates were then marked with Picard-Tools and Samtools was used to create a bcf-file containing the genomic positions. Variants were called and filtered with Bcftools using a minimum coverage of 10 and a quality threshold of 10.

### Genome comparison

For better comparison of both genomes, the *Trichoplax* reference genome was cut-off below 2 kb and cleaned from endosymbiont sequences (see above). This procedure resulted in 703 scaffolds amounting to 104.6 Mb of which 10.8 Mb are Ns.

On the nucleotide level, the *Trichoplax* sp. H2 genome was aligned to the *Trichoplax adhaerens* genome with LAST 749^77^ using lowercase masking of simple repeats and the subset seed NEAR for very closely related genomes. Lastal was then run with -m100, E0.05 and piped into last-split with -m1 to align each basepair of the H2 genome only once. Alignment statistics were calculated using the Last maf-convert script, the tool MafFilter^78^ and LibreOffice Calc.

For synteny analyses based on gene models generated by Maker, the SynMap pipeline at CoGe (genomevolution.org)^79^ was used, implementing LAST for finding best protein pairs, DAGchainer^80^ for identification of collinear pairs and CodeML^81^ for the calculation of pairwise synonymous and non-synonymous substitution rates. Genomic regions were considered syntenic between the two genomes if they harbored at least five collinear pairs allowing a maximum distance of 20 intervening genes. The synonymous and non-synonymous substitution rates between collinear CDS pairs of the two placozoan genomes were calculated with CodeML^81^. Values for dS and dN of 2 or more were considered saturated and excluded for further calculations. dN/dS ratios were only calculated if dN or dS had values above zero. The ratios were log10 transformed and binned into 60 size categories with Gnumeric. Synteny analyses were also performed using SyMAP v4.2^82^ with default parameters. Both analysis pipelines were provided with the Maker generated GFF.

Orthologous clustering between the Maker generated gene models from *Trichoplax* sp. H2 and *Trichoplax adhaerens* was done using Orthovenn^83^. The single copy orthologs were then compared with BLASTP to calculate overall protein identity. Because gene models differ to some extent even between closely related species, which is even more pronounced in an evidence-based gene prediction, the BLASTP output was cleaned from alignments where the length difference between pairs was more than 10% of a pair’s average length and the BLASTP alignment length deviated from pair average length more than 10%.

### Phasing of representative coding sequences

Since both genome assemblies represent the un-phased consensus of two alleles, it was tried to reconstruct these for the coding sequences (CDS) of representative genes in order to answer the question if the observed polymorphisms between the two lineages could be the result of polymorphisms within them. The CDS were chosen because comparable datasets for both lineages were available as two paired-end RNA-Seq datasets.

For this purpose, thirty CDS pairs were chosen that showed at least one SNP between the two lineages on either genomic or transcriptomic CDS. We chose a mix of genes that consisted of highly-expressed genes (e.g. like Tubulin beta), genes of general interest (e.g. like several transcription factors) or genes that were conspicuous by their high dN/dS ratio (e.g. DBX, NK6). These genes were insofar randomly chosen as we had no prior knowledge about their phasing. They are furthermore representative for both genomes because they are located on 10 and 28 different scaffolds in the genome assemblies of *Trichoplax adhaerens* and *Trichoplax* sp. H2, respectively. All CDS were taken from the two assembled transcriptomes to avoid discrepancies between gene models. The only exception was the CDS of the placozoan NK6 ortholog which was found to be fragmented in the *Trichoplax* sp. H2 transcriptome as a result of the lower coverage. It was therefore replaced by the genomic prediction which is identical in size to the H1 genomic and transcriptomic predictions.

Phasing was performed by mapping the quality trimmed paired-end RNA-Seq reads against the CDS using the Geneious mapper (Geneious 8.1^84^) and carefully tracing the overlapping reads and their mates from SNP to SNP in the Geneious browser by eye. Because the insert size and read length of the Illumina libraries was sometimes not sufficient to bridge larger distances between two adjacent SNPs, some CDS could not be phased into a single block with the RNA-Seq data alone. To further merge multiple phased blocks per CDS, the genomic paired-end reads of *Trichoplax* sp. H2 and the *Trichoplax adhaerens* trace reads^85^ were therefore mapped against the annotated respective genomic loci. Potential artifacts due to sequencing errors can be excluded since half of the investigated transcripts had a mean read coverage of 1,000x or more and the remaining transcripts’ coverage ranged from 35x to 800x, with the exception of the two NK6 orthologs (9x in H2, 23x in H1; see Supplementary Table S4 for mean read coverage of all transcripts). The low RNA-seq read coverage for NK6 nevertheless allowed the identification of SNPs and these could be further verified by the genomic reads (H2) or genomic trace reads (H1), respectively.

### Data availability

The annotated Whole Genome Shotgun project of *Trichoplax* sp. H2 has been deposited at DDBJ/ENA/GenBank under the accession NOWV00000000. The version described in this paper is version NOWV01000000. Individual genes or products described in this paper are indicated by their locus_tag. The Trinity Transcriptome Shotgun Assembly projects of *Trichoplax* sp. H2 and *Trichoplax adhaerens* have been deposited at DDBJ/EMBL/GenBank under the accessions GFSF00000000 and GFSG00000000, respectively. The versions described in this paper are the first versions, GFSF01000000 and GFSG01000000. Individual transcripts (e.g. used for transcript phasing) are indicated by their sequence names. Genomic Paired-End Illumina reads of *Trichoplax* sp. H2 have been deposited at the NCBI Sequence Read Archive under the accessions SRR5934055 (150 bp reads). Illumina Paired-End RNA-seq reads of *Trichoplax* sp. H2 and *Trichoplax adhaerens* have been deposited at the NCBI Sequence Read Archive under the accessions SRR5819939 and SRR5826498, respectively. The cleaned and re-annotated genome of *Trichoplax adhaerens* (source JGI: http://genome.jgi.doe.gov/Triad1/Triad1.home.html) has been deposited at the CoGe Comparative Genomics website (https://www.genomevolution.org/coge/) under the genome ID 31909.

## Electronic supplementary material

Supplementary Information

Dataset 1

Dataset 2

Dataset 3

Dataset 4
