# Supplementary material for: Trichoplax genomes reveal profound admixture and suggest stable wild populations without bisexual reproduction
Source: Sci Rep. 2018 Jul 24;8:11168. doi: 10.1038/s41598-018-29400-y (PMC6057997; doi:10.1038/s41598-018-29400-y)
Supplement: Supplementary file 1 — Supplementary Information [file 41598_2018_29400_MOESM1_ESM.pdf]

Trichoplax genomes reveal profound admixture and suggest stable wild populations without bisexual reproduction

Kai Kamm*, Hans-Jürgen Osigus, Peter F. Stadler, Rob DeSalle, Bernd Schierwater

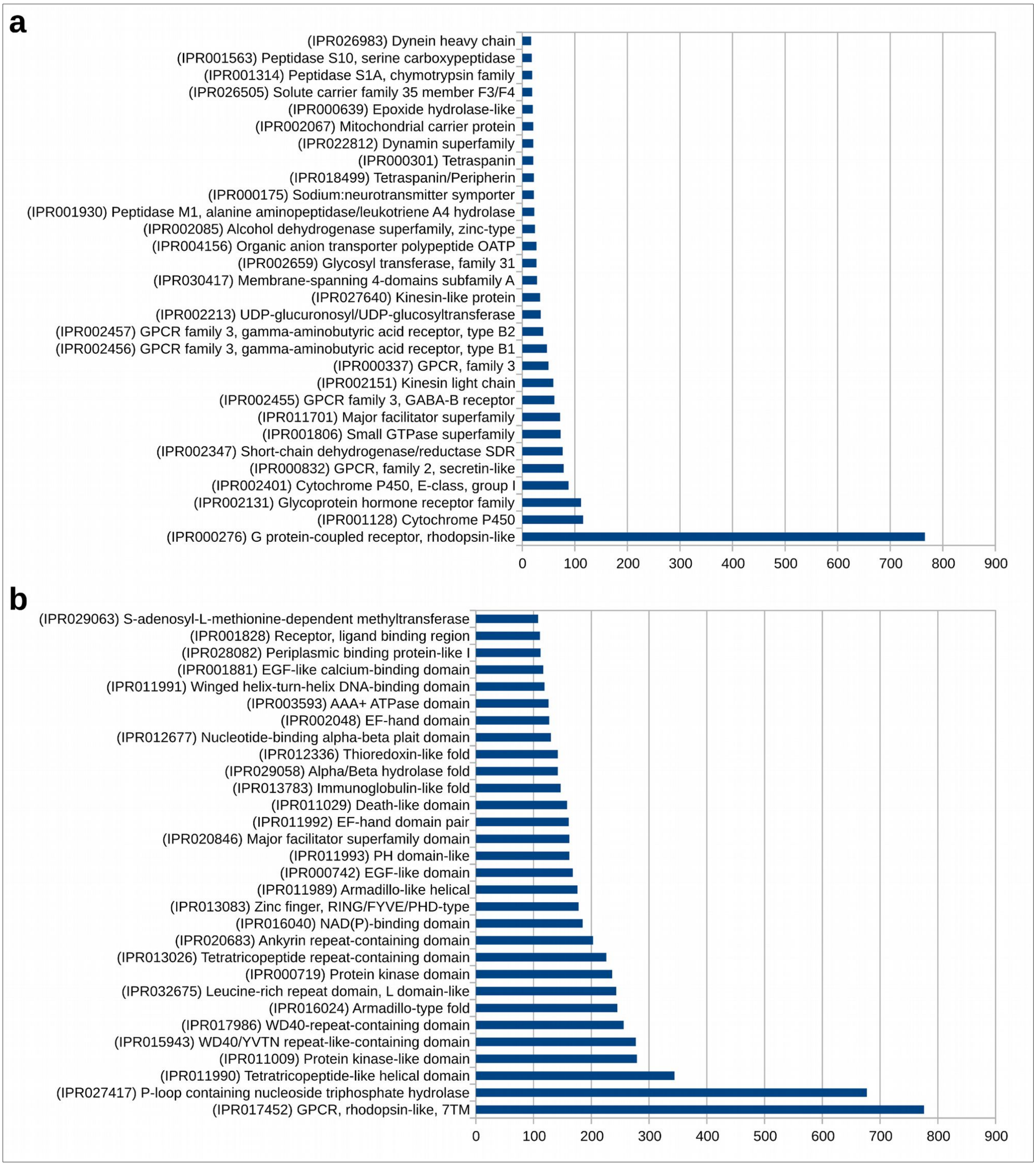

Supplementary Figure S1: Summary of the functional annotation of the *Trichoplax* sp. H2 genome.

(**a**) The thirty most abundant InterPro families. (**b**) The thirty most abundant InterPro domains.

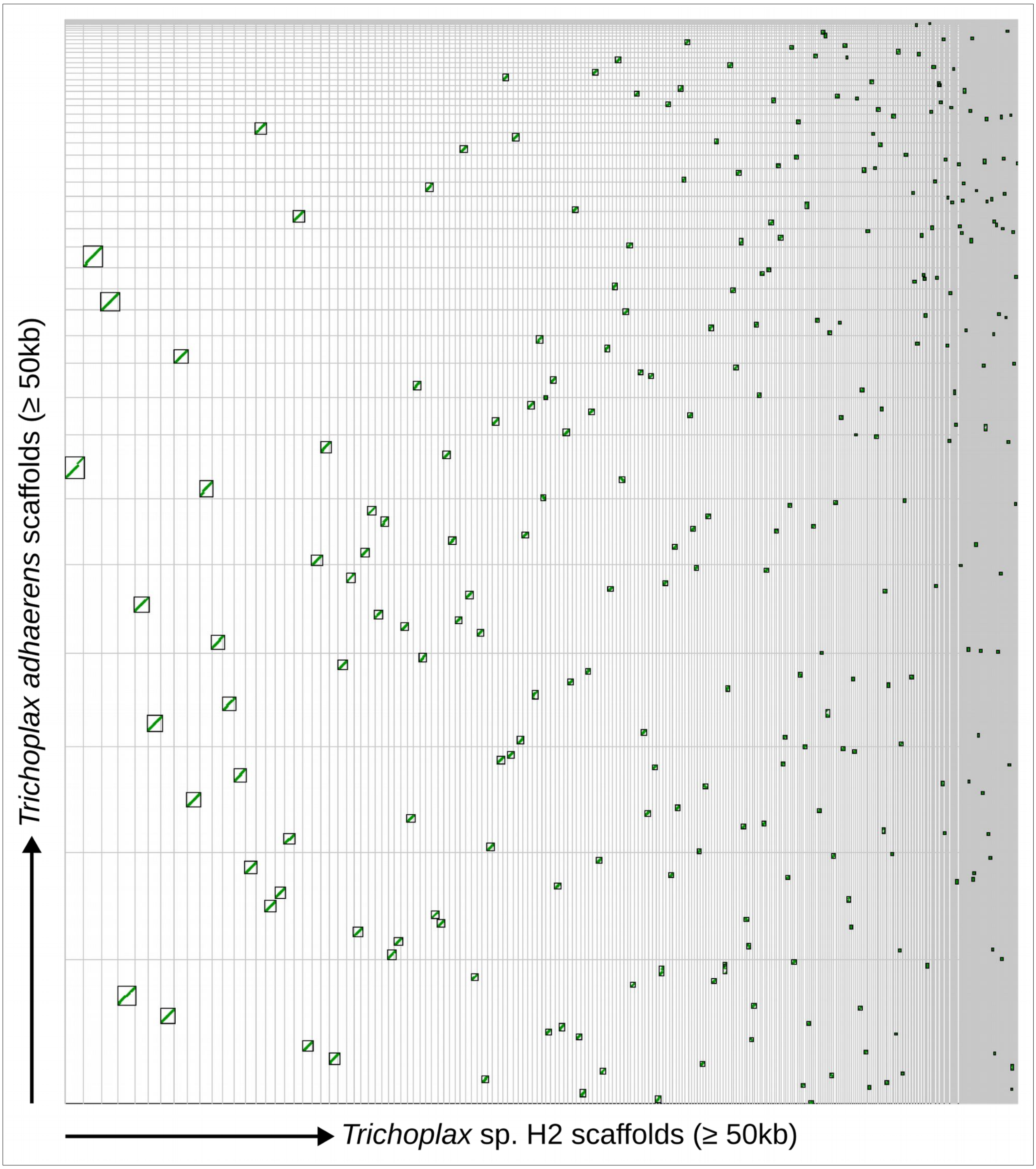

Supplementary Figure S2: Comparison of the genomic organization

Dot-blot of the syntenic regions between the genome assemblies of *Trichoplax adhaerens* and *Trichoplax* sp. H2 based on gene-models. Green dots surrounded by boxes fulfill criteria of synteny. For clarity only scaffolds of at least 50kb are included.

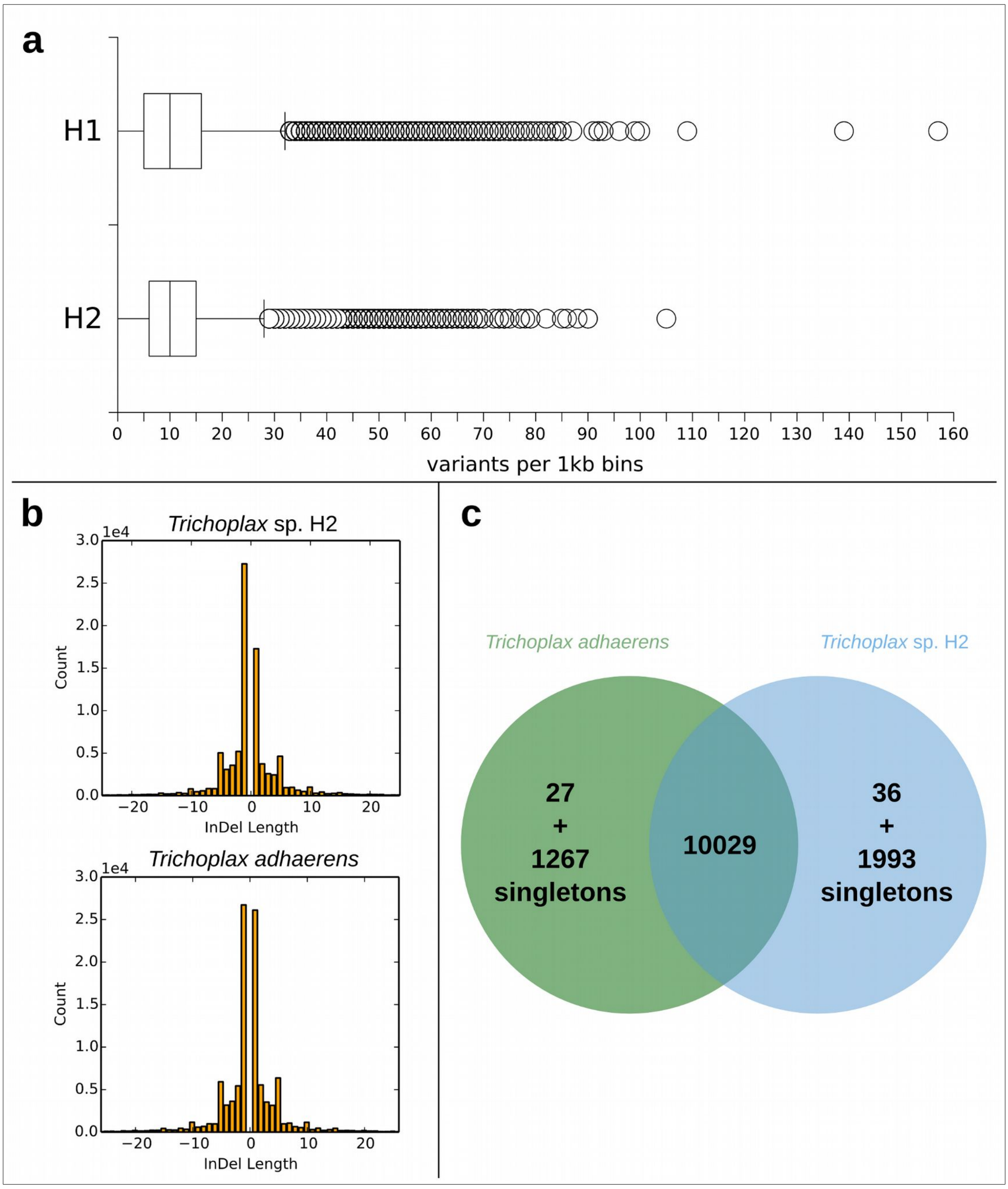

Supplementary Figure S3: Variant analyses of the two *Trichoplax* genomes and orthologous clustering of gene models

(**a & b**) Variant analyses of the two genomes using the genomic reads of *Trichoplax* sp. H2 for both genomes. (**a**) Boxplot showing single nucleotide polymorphisms (SNPs) per 1kb in the two genomes (box width = interquartile range, vertical line within box = median, circles = outliers, whiskers extend 1.5 times the interquartile range in both directions of the box' margin). (**b**) Count and length distribution of InDels. (**c**) Orthologous clustering of *Trichoplax adhaerens* and *Trichoplax* sp. H2 gene models. The two lineages share more than 10,000 orthologous clusters but possess a certain amount of singletons, possibly as a result of a combination of 1. differences in the calling of the evidence-based gene models 2. non-overlapping portions of the respective assemblies 3. lineage specific genes.

**Hhex**	711bp									
scaffold_17		138	246	262	273	331	419	422	618	621
H1		G	T	C	G	G	T	G	G	C
H1	*	G	C	T	A	A	T	A	A	T
H2	*	G	C	T	A	A	T	A	A	T
H2		A	T	C	G	G	G	G	G	C
scaffold_37										

**NK6**	651bp						**HD**			
scaffold_17		52	69	188	226	281	504	520	530	635
H1		G	C	T	T	G	C	C	G	G
H1	*	A	C	T	C	A	T	C	A	G
H2	*	A	C	T	C	A	T	C	A	G
H2		A	T	C	T	G	C	G	A	A
scaffold_37										

**Bmp7**	1134bp		**TGFb_propeptide**												**TGF_beta**	
scaffold_7		47	150	400	457	458	544	577	616	643	652	756	779	802	1043	1059
H1		T	C	A	A	A	T	A	A	C	G	A	A	A	C	T
H1		C	T	A	T	C	C	C	G	A	G	G	G	G	G	C
															TGA	
H2		C	T	A	T	C	C	C	G	A	G	G	G	G	C	C
H2		T	C	G	A	A	C	C	A	C	A	A	A	A	C	T
scaffold_22																

**PRKAG2**	1287bp			**CBS**			**CBS**				**CBS**		
scaffold_3		222	257	549	597	723	775	837	1063	1099	1163	1212	1283
H1	#	G	A	A	T	T	G	T	T	G	G	C	T
H1	*	A	G	G	A	C	A	C	C	T	A	A	A
H2	*	A	G	G	A	C	A	C	C	T	A	A	A
H2	#	G	A	A	T	T	G	T	T	G	G	C	T
scaffold_13													

Supplementary Figure S4: Phasing of representative transcripts

Four examples of the allele comparison between *Trichoplax adhaerens* and *Trichoplax* sp. H2. Shown are the SNP positions of the phased CDS. A continuous color denotes a single phased block. Identical alleles are marked with * or # and non-synonymous SNPs are framed by black boxes. If a SNP position falls into the region of a conserved domain this is indicated by a grey bar above. Note that the grey bar does not reflect exact length or position of the conserved domain within the complete CDS/protein. In the case of the placozoan bone morphogenetic protein (BMP) the two closest alleles between the two lineages differ in only one substitution (position 1043) which introduced a stop codon (red) in the H1 allele and cuts off the last third of the TGF-beta domain. Complete results and accessions for the 30 CDS are given in Supplementary Table S4.

Supplementary Tables (as separate spreadsheets):

Supplementary Table S1: Statistics of the genome/transcriptome assembly and annotation

Genome annotation statistics, transcriptome assembly summary, variant calling summary, examples of novel placozan genes, KEGG mapping results.

Supplementary Table S2: Repeat analyses and comparison of the two placozoan genomes

Supplementary Table S3: Examples of *Trichoplax* sp. H2 CDS showing dN/dS values above 1.5

Supplementary Table S4: Details and accessions of the phased CDS of *Trichoplax* sp. H2 and *Trichoplax adhaerens*
